# Block-Iterative Reconstruction from Dynamically Selected Sparse Projection Views Using Extended Power-Divergence Measure

**DOI:** 10.3390/e24050740

**Published:** 2022-05-23

**Authors:** Kazuki Ishikawa, Yusaku Yamaguchi, Omar M. Abou Al-Ola, Takeshi Kojima, Tetsuya Yoshinaga

**Affiliations:** 1 Graduate School of Health Sciences, Tokushima University, 3-18-15 Kuramoto, Tokushima 770-8509, Japan; c202026001@tokushima-u.ac.jp; 2 Shikoku Medical Center for Children and Adults, National Hospital Organization, 2-1-1 Senyu, Zentsuji 765-8507, Japan; yamaguchi.yusaku.sf@mail.hosp.go.jp; 3 Faculty of Science, Tanta University, El-Giesh St., Tanta 31527, Gharbia, Egypt; omar26_7@yahoo.com; 4 Institute of Biomedical Sciences, Tokushima University, 3-18-15 Kuramoto, Tokushima 770-8509, Japan; kojima@medsci.tokushima-u.ac.jp

**Keywords:** power-divergence measure, computed tomography, iterative reconstruction, ordered-subsets algorithm, block-iterative reconstruction

## Abstract

Iterative reconstruction of density pixel images from measured projections in computed tomography has attracted considerable attention. The ordered-subsets algorithm is an acceleration scheme that uses subsets of projections in a previously decided order. Several methods have been proposed to improve the convergence rate by permuting the order of the projections. However, they do not incorporate object information, such as shape, into the selection process. We propose a block-iterative reconstruction from sparse projection views with the dynamic selection of subsets based on an estimating function constructed by an extended power-divergence measure for decreasing the objective function as much as possible. We give a unified proposition for the inequality related to the difference between objective functions caused by one iteration as the theoretical basis of the proposed optimization strategy. Through the theory and numerical experiments, we show that nonuniform and sparse use of projection views leads to a reconstruction of higher-quality images and that an ordered subset is not the most effective for block-iterative reconstruction. The two-parameter class of extended power-divergence measures is the key to estimating an effective decrease in the objective function and plays a significant role in constructing a robust algorithm against noise.

## 1. Introduction

The iterative reconstruction [1,2] of density pixel images from measured projections in computed tomography [3,4,5,6,7] has attracted considerable attention, in spite of its high computational cost because of its ability to produce a higher-quality image compared with transform methods [8,9]. For compensating the drawback of the slow convergence of the simultaneous iterative image reconstruction algorithm [10,11,12,13], projections can be divided up into multiple blocks or subsets. The ordered-subsets (OS) algorithm [10,12,14,15] is an acceleration scheme that uses subsets in a previously decided order. There exist degrees of freedom in not only the number of divisions but also the order of subsets with which to obtain an accelerated OS algorithm that can perform a high-image-quality reconstruction quickly. Several methods have been proposed to improve convergence rate by permuting the order of projections; for example, fixed angle (FAS), prime number decomposition (PND) [16], random access (RAS) [17,18], multilevel access (MLS) [19], and weighted distance (WDS) [20] are constructed such that the projection access space sampling is as uniform as possible. However, they do not incorporate object information, such as shape, into the selection process.

The purpose of this study is to construct an object-dependent rule for selecting more effective subsets to accelerate convergence. It shows that an algorithm with dynamically selected projection views at every iteration, rather than ordered projections, is a more effective scheme from the viewpoint of total optimization. The proposed procedure, which is an unordered-subsets (unOS) algorithm, selects the subset index that gives the largest decrease in the objective function between at the initial value and at one-step later by using a subset selected according to a function value estimated from the initial value. Block-iterative (BI) algorithms of the simultaneous algebraic reconstruction technique (SART) [1], maximum-likelihood expectation-maximization (MLEM) [21], and the simultaneous multiplicative algebraic reconstruction technique (MART) [2,22,23] are applicable to the proposed selection or estimating scheme. The term BI in this paper has the same meaning as OS, except for whether the projections are ordered or not. As a theoretical basis of the proposed strategy, we prove a unified proposition on inequalities related to the difference between the objective functions caused by a one-step iteration by using an extended power-divergence measure [24,25,26,27], which includes a wide set of known distance and relative entropy measures with two variable parameters. When the number of subsets is the same as the number of projections, the decrease in the difference between the objective functions is identical to the value of the estimating function, and, therefore, the selection of the subset for which the estimating function has the largest value gives a more effective optimization.

We conducted experiments on tomographic image reconstruction from sparse projection views with a relatively large number of subsets compared with the projection number and obtained results showing a high satisfaction rate of the desired subset relation through use of the estimating function supported by a theoretical analysis. Practically speaking, although the computation time of the estimating function is an issue, a fast matrix-vector multiplication can mitigate this problem, and its effect is worth the delay, as illustrated in the experiment.

## 2. Problem Description

### 2.1. Block Iterative Reconstruction

Image reconstruction is the problem of obtaining unknown pixel values $x\in R_{+}^{J}$ satisfying

(1) y=Ax+δ

where $y\in R_{+}^{I}$, $A\in R_{+}^{I\times J}$, and $\delta \in R^{I}$ denote the measured projection, projection operator, and noise, respectively, with $R_{+}$ representing the set of nonnegative real numbers. When the system in Equation (1) without noise, i.e., δ=0, has a solution e∈E with

(2) $$E:=\left\{e\in R_{+}^{J}\;|\;y=Ae\right\},$$

it is consistent; otherwise, it is inconsistent. The inverse problem of tomography can be reduced to one of finding *x*, which can be accomplished by using an optimization approach such as an iterative method minimizing an objective function.

**Figure 1:**
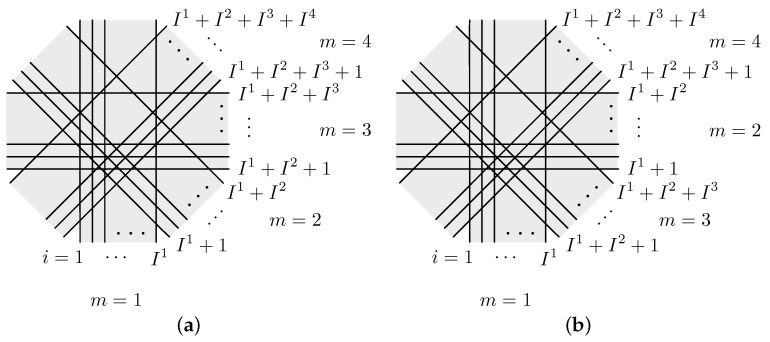
Access scheme for projections $y_{i}$, $i=1,2,\ldots ,-I^{1}+I^{2}+I^{3}+I^{4}$ with M=4 in (**a**) SAS and (**b**) MLS.

For formulating BI algorithms, let $y^{m}\in R_{+}^{I^{m}}$ and $A^{m}\in R_{+}^{I^{m}\times J}$ be, respectively, a subvector consisting of $I^{m}$ partial elements of *y* and a submatrix of *A* with the same corresponding rows of $y^{m}$ for m=1,2,…,M, where *M* indicates the total number of divisions or the subset number. Figure 1 shows an example of the projection access scheme in the order of sequential access (SAS), which is the natural access order of the acquired projections, and MLS for M=4.

### 2.2. Preliminaries

Here, we introduce the notation that will be used below. The superscript ⊤ stands for the transpose of a matrix or vector, $\theta_{k}$ indicates the *k*th element of the vector θ, $\Theta_{i}$ and $\Theta_{ij}$ indicate the *i*th row vector and the element in the *i*th row and *j*th column of the matrix Θ.

The generalized Kullback–Leibler (KL) divergence or relative entropy is defined as

(3) $$\mathrm{KL}\left(p,q\right):=\sum_{k}p_{k}\log\frac{p_{k}}{q_{k}}+q_{k}-p_{k}=\sum_{k}\int_{p_{k}}^{q_{k}}\frac{s-p_{k}}{s}ds$$

for given nonnegative vectors *p* and *q*. The divergence KL(p,q), known as the Csiszár’s *I*-divergence measure [28], is nonnegative with KL(p,q)=0 if and only if p=q. Moreover, we let ${\mathrm{EP}}_{\gamma ,\alpha }\left(p,q\right)$ be a parameterized estimating function of nonnegative vectors *p* and *q*,

(4) $${\mathrm{EP}}_{\gamma ,\alpha }\left(p,q\right):=\sum_{k}\int_{p_{k}}^{q_{k}}\frac{s^{\gamma }-p_{k}^{\gamma }}{s^{\gamma \alpha }}ds$$

where γ and α, respectively, indicate positive and nonnegative parameters, which is a two-parameter extension [29] of the power-divergence measure and coincides with the KL-divergence if (γ,α)=(1,1), the squared $L^{2}$ norm if (γ,α)=(1,0), and so on.

Lastly, we define

(5) $$\lambda_{j}^{m}:={\left(\sum_{i=1}^{I^{m}}A_{ij}^{m}\right)}^{-1}$$

for j=1,2,…,J and let $\rho^{m}$ be the largest eigenvalue of the matrix ${A^{m}}^{⊤}A^{m}$ for m=1,2,…,M.

## 3. Results

This section describes the proposed method, theory, and optimization strategy. In the following, the term *weeding* will be used to refer to discarding subsets appearing in the proposed unOS scheme by likening them to weeds, as shown in the frequency bar chart of subset indices in the experiment below.

### 3.1. Proposed Method

We present unOS iterative algorithms, called the weeding BI reconstruction (WBIR) algorithms, for obtaining the pixel value z(n) as a function of the iteration number *n*, described by

(6) $$z_{j}\left(n+1\right)=z_{j}\left(n\right)+\frac{W^{m}\left(z\left(n\right)\right)}{\rho^{m}}\sum_{i=1}^{I^{m}}A_{ij}^{m}\left(y_{i}^{m}-A_{i}^{m}z\left(n\right)\right),$$

(7) $$z_{j}\left(n+1\right)=z_{j}\left(n\right)\exp\left(W^{m}\left(z\left(n\right)\right)\log\left(\lambda_{j}^{m}\sum_{i=1}^{I^{m}}A_{ij}^{m}\frac{y_{i}^{m}}{A_{i}^{m}z\left(n\right)}\right)\right),$$

and

(8) $$z_{j}\left(n+1\right)=z_{j}\left(n\right)\exp\left(W^{m}\left(z\left(n\right)\right)\lambda_{j}^{m}\sum_{i=1}^{I^{m}}A_{ij}^{m}\log\frac{y_{i}^{m}}{A_{i}^{m}z\left(n\right)}\right)$$

for *j* = 1, 2, …, *J*, *n* = 0, 1, 2, …, and *m* = (*n* mod *M*) +1 with *z*(0) = *z*^0^, where the function $W^{m}\left(z\left(n\right)\right)$, say the weeding function, takes either 0 or 1 and is defined for some γ and α by

(9) $$W^{m}\left(x\right):=U\left(\frac{{\mathrm{EP}}_{\gamma ,\alpha }\left(y^{m},A^{m}x\right)}{\max_{k}{\mathrm{EP}}_{\gamma ,\alpha }\left(y^{k},A^{k}x\right)}-\mu \right),\;x\notin E$$

with μ being a nonnegative parameter and U denoting the unit step function where U(θ)=0 for θ<0 and 1 for θ≥0.

Here, Equations (6), (7), and (8) are, respectively, equivalent to the OS-type BI-SART, BI-MLEM, and BI-MART algorithms, when the values of the function $W^{m}$ are identical to one by taking μ=0. The relaxation parameter $1/\rho^{m}$ in the BI-SART algorithm is chosen in order to make the iterations converge as rapidly as possible [15].

### 3.2. Theory

This section provides theoretical results on the systems defined in Equations (6)–(8) with μ=0 for a consistent tomographic inverse problem.

The first system considered here is the BI-SART algorithm.

**Proposition** **1.** *For e∈E and a solution z to the system in Equation (6) with μ=0, the inequality*,

(10) $$\|e-z{\left(0)\right\|}_{2}^{2}-\|e-z{\left(1)\right\|}_{2}^{2}\geq \frac{1}{\rho^{m}}\|y^{m}-A^{m}z{\left(0)\right\|}_{2}^{2},$$

*is satisfied for any m=1,2,…,M*.

In this Proposition and later, to simplify the description, the iteration numbers 0 and 1 are treated as *n* and n+1, respectively, for any given *m* and series of n=m−1,m,m+1,…, since the autonomous difference system is invariant to a discrete time shift, which means that the shift has no effect on the dynamics.

**Proof.**  As a special case described in Byrne [15], we have

(11) $$\begin{array}{ccc} \; & \; & \|e-z{\left(0)\right\|}_{2}^{2}-\|e-z{\left(1)\right\|}_{2}^{2}\\ \; & = & \frac{2}{\rho^{m}}{\left(e-z\left(0\right)\right)}^{⊤}{A^{m}}^{⊤}\left(y^{m}-A^{m}z\left(0\right)\right)-\|\frac{1}{\rho^{m}}{A^{m}}^{⊤}{\left(y^{m}-A^{m}z\left(0\right))\right\|}_{2}^{2}\\ \; & \geq & \frac{2}{\rho^{m}}\|y^{m}-A^{m}z{\left(0)\right\|}_{2}^{2}-\frac{1}{\rho^{m}}\|y^{m}-A^{m}z{\left(0)\right\|}_{2}^{2}\\ \; & = & \frac{1}{\rho^{m}}\|y^{m}-A^{m}z{\left(0)\right\|}_{2}^{2} \end{array}$$

□

**Corollary** **1.** *Under the assumption of Proposition 1 and when M = I, equality*

(12) $$\|e-z{\left(0)\right\|}_{2}^{2}-\|e-z{\left(1)\right\|}_{2}^{2}=\frac{1}{\rho^{m}}{\left(y^{m}-A^{m}z\left(0\right)\right)}^{2}$$

*is satisfied form* = 1, 2, …, *I*.

**Proof.**  For a scalar $y^{m}$ and a vector $A^{m}$, equality holds under $\rho^{m}=A^{m}{A^{m}}^{⊤}$ for any *m*. □

Now, let us consider the iterative algorithms of BI-MLEM and BI-MART.

**Proposition** **2.** *For e∈E and a solution z to each system in Equations (7) and (8) with μ=0, the inequality*,

(13) $$\sum_{j=1}^{J}{\left(\lambda_{j}^{m}\right)}^{-1}\mathrm{KL}\left(e_{j},z_{j}\left(0\right)\right)-\sum_{j=1}^{J}{\left(\lambda_{j}^{m}\right)}^{-1}\mathrm{KL}\left(e_{j},z_{j}\left(1\right)\right)\geq \mathrm{KL}\left(y^{m},A^{m}z\left(0\right)\right),$$

*is satisfied for any m=1,2,…,M*.

**Proof.**  Inequality (13) for the BI-MLEM algorithm in Equation (7) with μ=0 is derived as follows.

(14) $$\begin{array}{ccc} \; & \; & \sum_{j=1}^{J}{\left(\lambda_{j}^{m}\right)}^{-1}\mathrm{KL}\left(e_{j},z_{j}\left(0\right)\right)-\sum_{j=1}^{J}{\left(\lambda_{j}^{m}\right)}^{-1}\mathrm{KL}\left(e_{j},z_{j}\left(1\right)\right)-\mathrm{KL}\left(y^{m},A^{m}z\left(0\right)\right)\\ \; & = & \sum_{j=1}^{J}{\left(\lambda_{j}^{m}\right)}^{-1}\left(e_{j}\log\frac{z_{j}\left(1\right)}{z_{j}\left(0\right)}+z_{j}\left(0\right)-z_{j}\left(1\right)\right)\\ \; & \; & -\sum_{i=1}^{I^{m}}\left(y_{i}^{m}\log\frac{y_{i}^{m}}{{\left(A^{m}z\left(0\right)\right)}_{i}}+{\left(A^{m}z\left(0\right)\right)}_{i}-y_{i}^{m}\right)\\ \; & = & \sum_{j=1}^{J}\sum_{i=1}^{I^{m}}A_{ij}^{m}e_{j}\log\left(\lambda_{j}^{m}\sum_{k=1}^{I^{m}}\frac{A_{kj}^{m}y_{k}^{m}}{{\left(A^{m}z\left(0\right)\right)}_{k}}\right)\\ \; & \; & -\sum_{i=1}^{I^{m}}y_{i}^{m}\log\frac{y_{i}^{m}}{{\left(A^{m}z\left(0\right)\right)}_{i}}\\ \; & \geq & \sum_{j=1}^{J}\sum_{i=1}^{I^{m}}A_{ij}^{m}e_{j}\lambda_{j}^{m}\sum_{k=1}^{I^{m}}A_{kj}^{m}\log\frac{y_{k}^{m}}{{\left(A^{m}z\left(0\right)\right)}_{k}}\\ \; & \; & -\sum_{i=1}^{I^{m}}y_{i}^{m}\log\frac{y_{i}^{m}}{{\left(A^{m}z\left(0\right)\right)}_{i}}\\ \; & = & 0. \end{array}$$

While Inequality (13) for the BI-MART algorithm in Equation (8) with μ=0 is a special case obtained by Byrne [15], it can be proved in an alternative way using the procedure of direct reduction:

(15) $$\begin{array}{ccc} \; & \; & \sum_{j=1}^{J}{\left(\lambda_{j}^{m}\right)}^{-1}\mathrm{KL}\left(e_{j},z_{j}\left(0\right)\right)-\sum_{j=1}^{J}{\left(\lambda_{j}^{m}\right)}^{-1}\mathrm{KL}\left(e_{j},z_{j}\left(1\right)\right)-\mathrm{KL}\left(y^{m},A^{m}z\left(0\right)\right)\\ \; & = & \sum_{j=1}^{J}{\left(\lambda_{j}^{m}\right)}^{-1}\left(e_{j}\log\frac{z_{j}\left(1\right)}{z_{j}\left(0\right)}+z_{j}\left(0\right)-z_{j}\left(1\right)\right)\\ \; & \; & -\sum_{i=1}^{I^{m}}\left(y_{i}^{m}\log\frac{y_{i}^{m}}{{\left(A^{m}z\left(0\right)\right)}_{i}}+{\left(A^{m}z\left(0\right)\right)}_{i}-y_{i}^{m}\right)\\ \; & = & \sum_{i=1}^{I^{m}}y_{i}^{m}\log\frac{y_{i}^{m}}{{\left(A^{m}z\left(0\right)\right)}_{i}}\\ \; & \; & -\sum_{j=1}^{J}\sum_{i=1}^{I^{m}}A_{ij}^{m}z_{j}\left(0\right)\exp\left(\lambda_{j}^{m}\sum_{k=1}^{I^{m}}A_{kj}^{m}\log\frac{y_{k}^{m}}{{\left(A^{m}z\left(0\right)\right)}_{k}}\right)\\ \; & \; & -\sum_{i=1}^{I^{m}}y_{i}^{m}\log\frac{y_{i}^{m}}{{\left(A^{m}z\left(0\right)\right)}_{i}}+\sum_{i=1}^{I^{m}}y_{i}^{m}\\ \; & \geq & \sum_{i=1}^{I^{m}}y_{i}^{m}-\sum_{j=1}^{J}\sum_{i=1}^{I^{m}}A_{ij}^{m}z_{j}\left(0\right)\lambda_{j}^{m}\sum_{k=1}^{I^{m}}\frac{A_{kj}^{m}y_{k}^{m}}{{\left(A^{m}z\left(0\right)\right)}_{k}}\\ \; & = & 0. \end{array}\;$$

□

**Corollary** **2.** *Under the assumption of Proposition 2 and when M=I, equality*

(16) $$\sum_{j=1}^{J}{\left(\lambda_{j}^{m}\right)}^{-1}\mathrm{KL}\left(e_{j},z_{j}\left(0\right)\right)-\sum_{j=1}^{J}{\left(\lambda_{j}^{m}\right)}^{-1}\mathrm{KL}\left(e_{j},z_{j}\left(1\right)\right)=\mathrm{KL}\left(y^{m},A^{m}z\left(0\right)\right)$$

*is satisfied for m=1,2,…,I*.

**Proof.**  When M=I or equivalently $I^{m}=1$, for a scalar $y^{m}$ and a vector $A^{m}$, we have $\lambda_{j}^{m}A_{1j}^{m}=1$ for any *j* and *m*; therefore, each of the nonstrict Inequalities (14) and (15) reduces to an equality. □

The inequalities appearing in Propositions 1 and 2 can be described in a unified formula using nonnegative functions as follows:

(17) $$D^{m}\left(e,z\left(0\right)\right)-D^{m}\left(e,z\left(1\right)\right)\geq \Psi^{m}\left(y^{m},A^{m}z\left(0\right)\right)$$

for m=1,2,…,M using

(18) $$W^{m}\left(x\right):=U\left(\frac{\Psi^{m}\left(y^{m},A^{m}x\right)}{\max_{k}\Psi^{k}\left(y^{k},A^{k}x\right)}-\mu \right),\;x\notin E$$

where

(19) $$D^{m}\left(a,b\right):=||a-{b||}_{2}^{2}$$

and

(20) $$\Psi^{m}\left(p,q\right):=\frac{1}{\rho^{m}}{\mathrm{EP}}_{1,0}\left(p,q\right)$$

for BI-SART in Equation (6) with μ=0 and

(21) $$D^{m}\left(a,b\right):=\sum_{j=1}^{J}{\left(\lambda_{j}^{m}\right)}^{-1}\mathrm{KL}\left(a_{j},b_{j}\right)$$

and

(22) $$\Psi^{m}\left(p,q\right):={\mathrm{EP}}_{1,1}\left(p,q\right)$$

for BI-MLEM and BI-MART in Equations (7) and (8) with μ=0. Similarly, when M=I, the equalities in Corollaries 1 and 2 can be written as

(23) $$D^{m}\left(e,z\left(0\right)\right)-D^{m}\left(e,z\left(1\right)\right)=\Psi^{m}\left(y^{m},A^{m}z\left(0\right)\right)$$

for any m=1,2,…,M in accordance with the definitions of Equations (18)–(22).

### 3.3. Optimization Strategy

For a given set of initial values $Z^{0}\subseteq R_{+}^{J}$, consider

(24) $$\begin{array}{cc} Z:=\{z\left(0\right)\in Z^{0}\;|\;\underset{m}{\mathrm{argmax}}\left(D^{m}\left(e,z\left(0\right)\right)-D^{m}\left(e,z\left(1\right)\right)\right) & \\ ⊇\underset{m}{\mathrm{argmax}}\left(\Psi^{m}\left(y^{m},A^{m}z\left(0\right)\right)\right) & \;\;\;\;\;\}. \end{array}$$

The predicate in the set definition means that an element *m* in the subset indices giving the largest value of the estimating function $\Psi^{m}$ results in the largest decrease in the objective function $D^{m}$ by a one-step update. Thus, the aim of the optimization is to find an unOS iterative algorithm such that Z is almost equal to $Z^{0}$. For this purpose, we choose μ=1 in Equation (18), which means that the state variable z(n) in Equations (6)–(8) at *m* = (*n* mod *M*) + 1 is updated if the value of $\Psi^{m}\left(y^{m},A^{m}z\left(n\right)\right)$ at the *m*th subset is the largest among $\Psi^{k}\left(y^{k},A^{k}z\left(n\right)\right)$ for all k=1,2,…,M under which $Z=Z^{0}$ can be expected to hold.

On the basis of Corollaries 1 and 2 and the unified description of the equality in Equation (23) with Equation (18) and Equations (19)–(22), we see that Z is equal to $Z^{0}$ for $Z^{0}=R_{+}^{J}$ when M=I. While, unfortunately, $Z=Z^{0}$ is not satisfied in general when M<I, the numerical experiments described in the proceeding section indicate that the satisfaction rate is probabilistically high. Namely, the lower bound of Inequality (17) is sharp enough to satisfy the predicate in Equation (24) most of the time. Therefore, we assert that the function $\Psi^{m}$ can be used for estimating an effective decrease in the objective function $D^{m}$.

## 4. Experiments and Discussion

Let us illustrate the above theory and the effectiveness of the proposed algorithms in Equations (6)–(8) with the weeding function in Equation (9) by using examples from numerical experiments.

We examined an experiment in which the iteration number

(25) $$N:=\sum_{n=0}^{T-1}W^{m}\left(z\left(n\right)\right)$$

with *m* = (*n* mod *M*) + 1 is constant for a given *T*, which is the maximum number of iterations, depending on μ. The weeding rate defined as

(26) $$R:=\left(1-\frac{N}{T}\right)\times 100\left[\%\right]$$

indicates the rate of discarding subsets. The value μ=0 results in R=0 for representing an ordinary OS algorithm.

Note that, in Equations (6)–(8), when $W^{m}\left(z\left(n\right)\right)=0$ for some *n* with *m* = (*n* mod *M*) + 1, we have $z_{j}\left(n+1\right)=z_{j}\left(n\right)$. Then, the calculation for updating $z_{j}$ for any *j* is not necessary at this iteration number, which can be neglected when counting the iterations. Hence, in the graph presentation for WBIR, the iteration numbers *n* will be replaced with distinct numbers $n^{\prime }=0,1,2,\ldots ,\max\left\{0,\nu \left(n\right)-1\right\},\ldots ,N-1$, where ν(n) is defined by the nonnegative integers

(27) $$\nu \left(n\right)=\sum_{k=0}^{n}W^{m}\left(z\left(k\right)\right)$$

with *m* = (*k* mod *M*) + 1 for n=0,1,2,…,T−1.

All algorithms were executed using a 3.5 GHz 8-core Intel Xeon processor and computing tools provided by MATLAB (MathWorks, Natick, MA, USA) and highly optimized libraries for matrix-vector multiplication.

### 4.1. Verification of Theory

**Figure 2:**
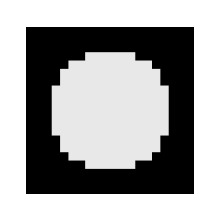
Phantom image *e* in 20×20 pixels.

**Figure 3:**
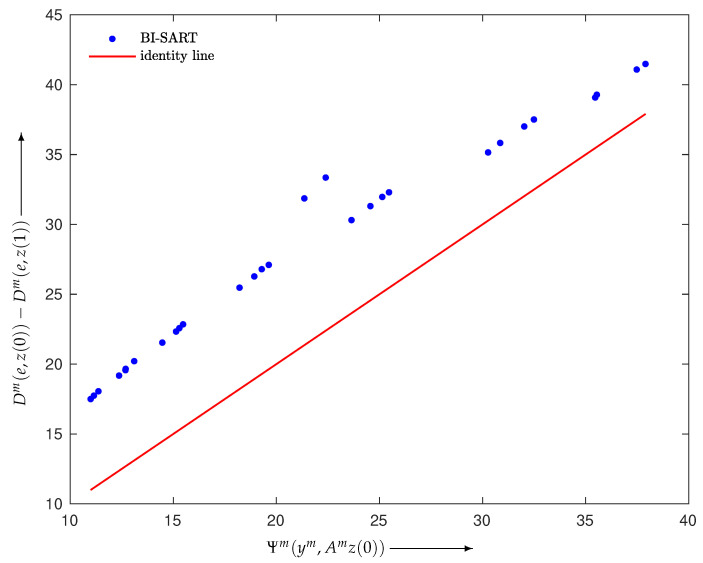
Scatter plot with identity line (red) for BI-SART in Equation (6).

**Figure 4:**
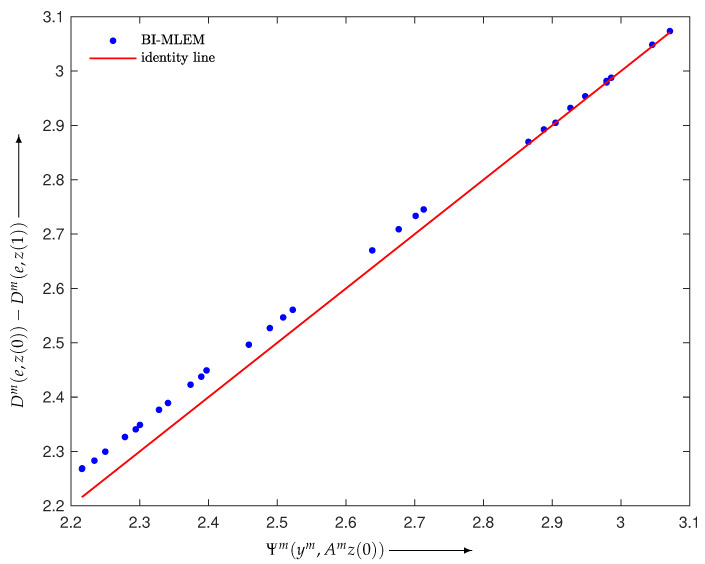
Scatter plot with identity line (red) for BI-MLEM in Equation (7).

**Figure 5:**
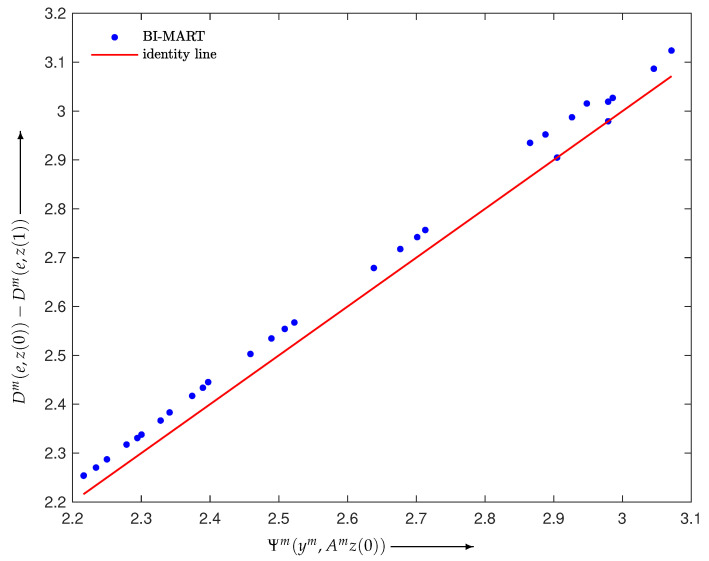
Scatter plot with identity line (red) for BI-MART in Equation (8).

Here, we give some results verifying Inequality (17). We defined the BI reconstruction algorithms as Equations (6)–(8) with $W^{m}\equiv 1$ and M=30 and constructed a noise-free projection $y=Ae\in R_{+}^{I}$ with I=930, where $e\in R_{+}^{J}$ with J=400 was made using the disc phantom shown in Figure 2. Examples verifying Inequality (17) are illustrated in Figure 3, Figure 4 and Figure 5, each of which plots

(28) $$\mathrm{RHS}\left(m\right):=\Psi^{m}\left(y^{m},A^{m}z\left(0\right)\right)$$

versus

(29) $$\mathrm{LHS}\left(m\right):=D^{m}\left(e,z\left(0\right)\right)-D^{m}\left(e,z\left(1\right)\right)$$

using a one-step iteration z(1) calculated from a given initial state z(0) with random elements for m=1,2,…,M. We can see that all *M* points are above the identity line, as stated in Propositions 1 and 2, and are located in their neighborhood.

We experimentally examined the set relation between $Z^{0}$ and Z in Equation (24). The elements of the sets ${\left\{\mathrm{LHS}(m)\right\}}_{m=1}^{M}$ and ${\left\{\mathrm{RHS}(m)\right\}}_{m=1}^{M}$ for the same initial value z(0) as above were sorted in descending order. The subset indices corresponding to the ten largest values are shown in the upper and lower rows in Table 1. We can see that both of the sets ${\mathrm{argmax}}_{m}\left(\mathrm{LHS}(m)\right)$ and ${\mathrm{argmax}}_{m}\left(\mathrm{RHS}(m)\right)$ are {17} for every BI reconstruction algorithm; thus, the relation of the predicate in Equation (24) or $Z=Z^{0}$ is satisfied in this example with $Z^{0}=\left\{z\left(0\right)\right\}$. For a large number of elements in $Z^{0}$, the rate at which the relation is satisfied is defined as the ratio of the number of elements in Z to that in $Z^{0}$. Experiments in which 100,000 independent trials with different seeds were used for generating the random elements of the initial values yielded 91.2%, 99.1%, and 86.7% satisfaction rates (in percentage) for the BI reconstruction algorithms in Equations (6), (7), and (8), respectively. These numerical simulation results show that the BI-MLEM almost always satisfies the set relation and therefore the MLEM-based WBIR has the best performance.

### 4.2. Evaluation of Reconstructed Images

**Figure 6:**
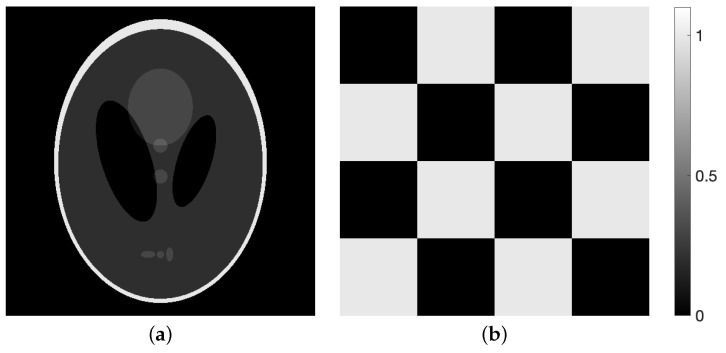
Phantom images of (**a**) Shepp–Logan and (**b**) chessboard pattern.

We verified the proposed strategy for the WBIR algorithm by comparing it with ordered-subsets expectation-maximization (OSEM) [10] with various projection access schemes in reconstruction experiments with practical sparse projection views. The base system of the WBIR was MLEM in Equation (7), which gave the highest satisfaction rate described above. We used either a Shepp–Logan or a chessboard pattern phantom image consisting of $e\in {\left[0,1\right]}^{J}$ with 512×512 pixels (J=262,144) (Figure 6). The projection $y\in R_{+}^{I}$ derived from the phantom image was simulated using Equation (1) without noise (δ=0), unless otherwise specified. The number of view angles, which were measured counterclockwise from a vertical line passing through the center of the phantom image, was set to 30 and the number of detector bins was set to 727, (I=21,810), with 180-degree sampling. We also set M=30, i.e., the same as the number of projection angles, for the BI algorithms and uniform initial values $z_{j}^{0}>0$ for j=1,2,…,J.

#### 4.2.1. Comparison with SAS-OSEM

**Figure 7:**
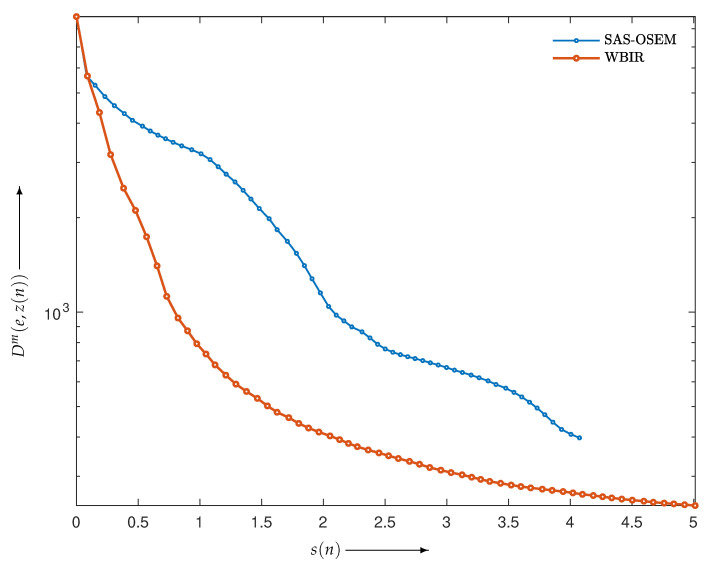
Objective functions $D^{m}\left(e,z\left(n\right)\right)$ for WBIR and conventional SAS-OSEM algorithms at each iteration n=0,1,2,…,60 in experiment using Shepp–Logan phantom.

We reconstructed the phantom image in Figure 6a by applying SAS-OSEM (short for OSEM with the projection access by SAS) and (MLEM-based) WBIR, i.e., Equation (7) with μ values of 0 and 1, respectively. Figure 7 is a graph of the objective function $D^{m}\left(e,z\left(n\right)\right)$ versus the real computation time s(n) (in seconds) for iteration numbers n=0,1,2,…,N where N=60. For every algorithm, the objective function monotonically decreases as the number of iterations increases. We can see that the WBIR algorithm reduces the objective function more than SAS-OSEM does. The reconstruction time of the SAS-OSEM algorithm is 4.1 s, whereas WBIR takes 5.0 s, both running 60 iterations. Although WBIR takes 20% longer than SAS-OSEM, it gives a smaller objective function when it reaches approximately the same computation time (s(48) for WBIR) as SAS-OSEM at the 60th iteration.

**Figure 8:**
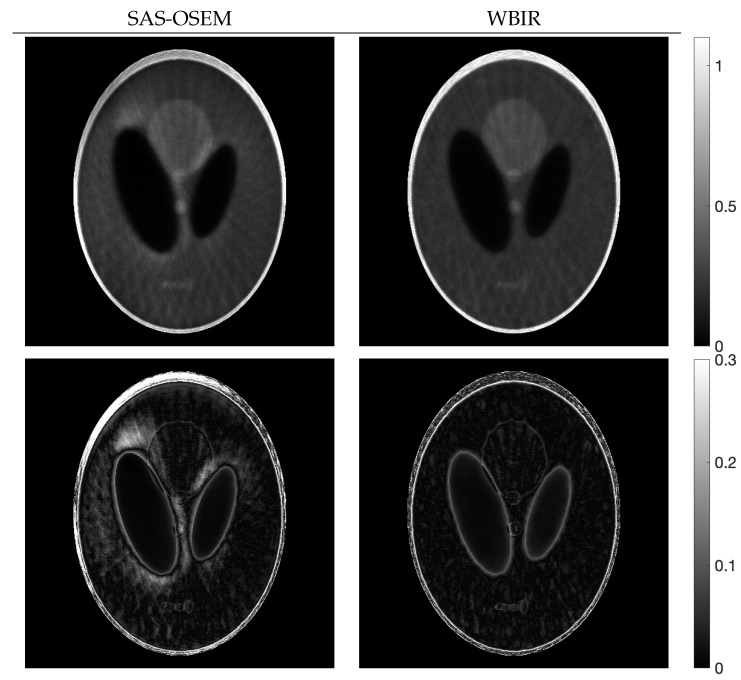
Reconstructed images (**upper** panel) and images of the subtraction (**lower** panel) for SAS-OSEM and WBIR in experiment using Shepp–Logan phantom.

Figure 8 illustrates images reconstructed with SAS-OSEM and WBIR at the 60th and 48th iterations, respectively, and the corresponding subtraction images at every *j*th pixel, defined as

(30) $${\left(d\left(z\right)\right)}_{j}:=\left|e_{j}-z_{j}\right|$$

for j=1,2,…,J. The density of *d* is on a common scale. By comparing the artifacts in the images, we can see that WBIR produces high-quality reconstructions, as is quantitatively indicated by the small measured objective function $D^{m}$ between the phantom and reconstructed images.

**Figure 9:**
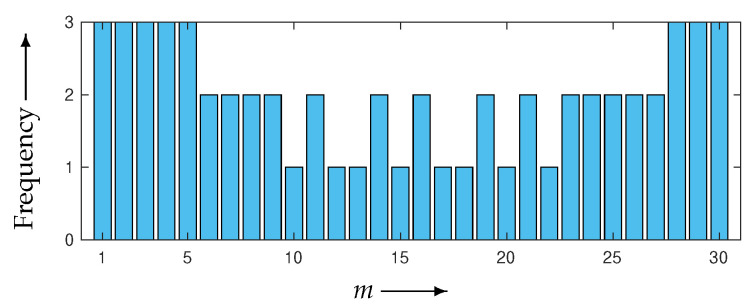
Frequency bar chart of subset indices m=1,2,…,30 for WBIR after 60 iterations in experiment using Shepp–Logan phantom.

The weeding rate *R* defined in Equation (26) for WBIR is 97%, by setting M=30 and μ=1. As shown in Figure 9, the frequency bar chart of the subset indices for WBIR is nonuniform, whereas that for OSEM is uniform. As far as we know, it is a novel property that a nonuniform and unordered (as opposed to uniform and ordered) use of projection views leads to higher-quality reconstructed images.

**Figure 10:**
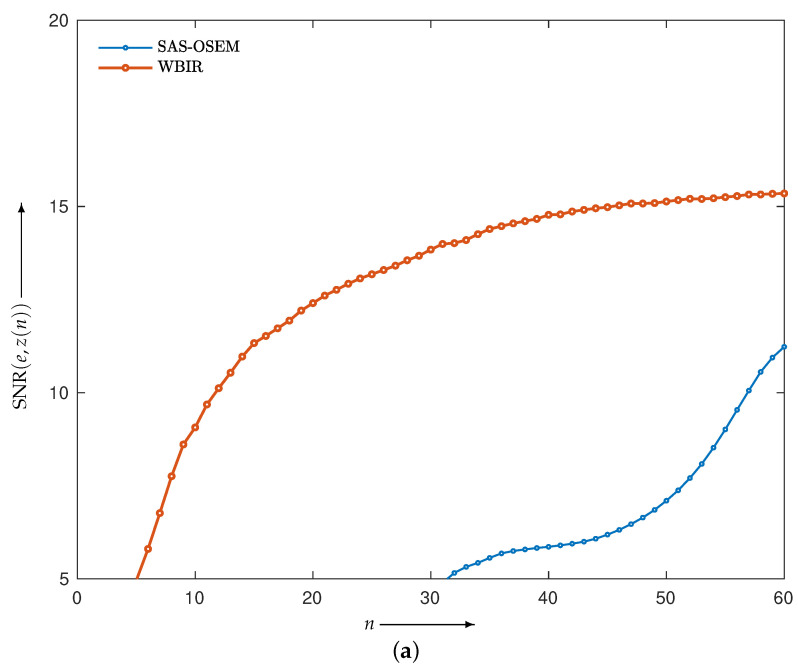

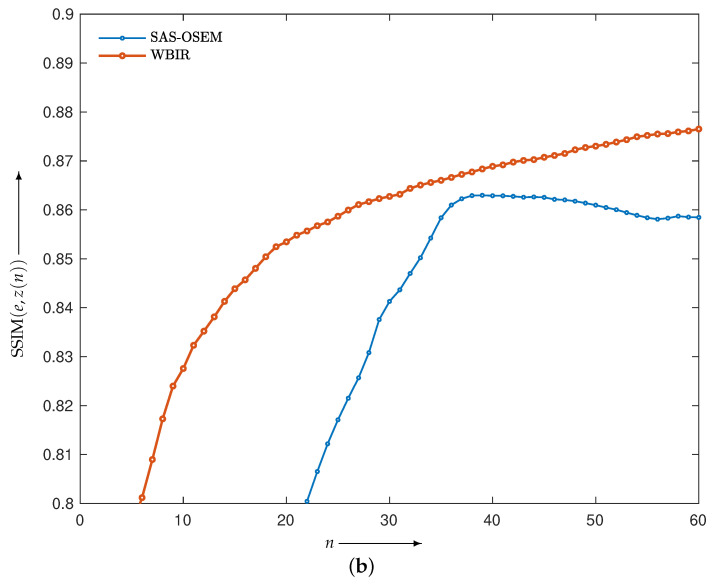
(**a**) SNR and (**b**) SSIM for WBIR and conventional SAS-OSEM algorithms at each iteration n=0,1,2,…,60 in experiment using Shepp–Logan phantom.

Besides an objective function, popular quantitative methods of evaluation include the signal-to-noise ratio (SNR(*e*,*z*(*n*))) and the structural similarity index measure (SSIM(*e*,*z*(*n*))), which is a perception-based quality metric, between the true image *e* and reconstructed image *z*(*n*). These were calculated for *n* = 0, 1, 2, …, 60 and are plotted in Figure 10a,b. Higher SNR and SSIM mean higher image quality. We can see that WBIR can reconstruct high-quality images with the same number of iterations while reducing artifacts caused by the sparse projections.

#### 4.2.2. Comparison with OSEM with Non-SAS

We experimentally compared the MLEM-based WBIR with OSEM algorithms using (deterministic) projection access schemes with PND, FAS, WDS, and MLS. The subset number *M* was fixed to 30 and the parameter μ controlling the weeding rate for WBIR was set to one.

**Figure 11:**
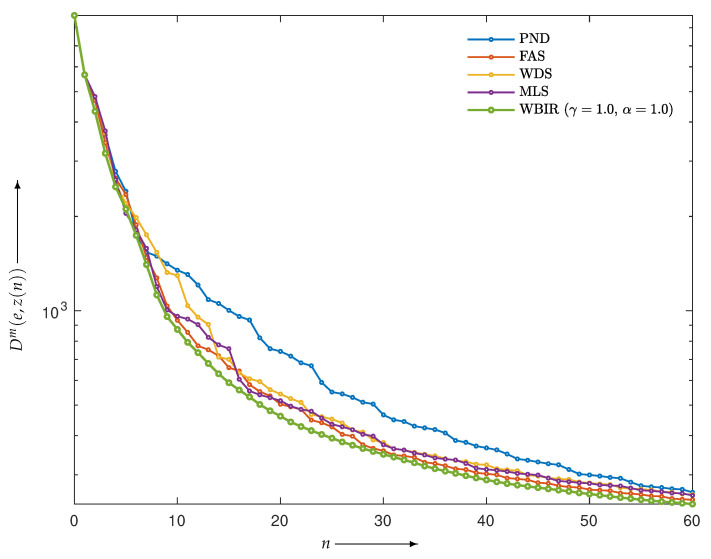
Objective functions $D^{m}\left(e,z\left(n\right)\right)$ for WBIR and OSEM algorithms by PND, FAS, WDS, and MLS at each iteration n=0,1,2,…,60 in experiment using Shepp–Logan phantom.

**Figure 12:**
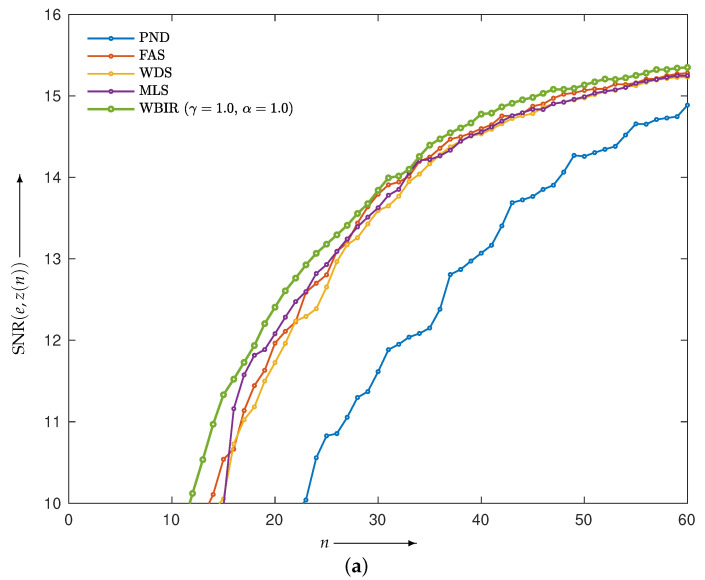

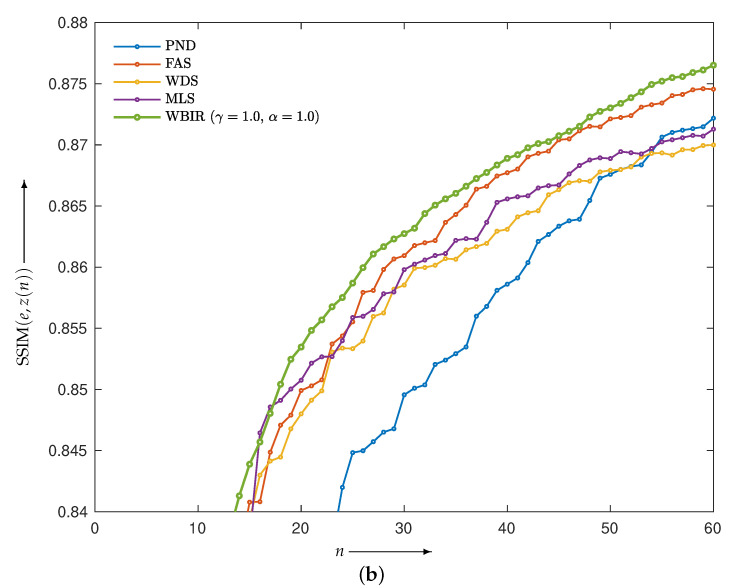
(**a**) SNR(*e*,*z*(*n*))) and (**b**) SSIM(*e*,*z*(*n*))) for WBIR and OSEM algorithms by PND, FAS, WDS, and MLS at each iteration *n* = 0, 1, 2, …, 60 in experiment using Shepp–Logan phantom.

First, we examined the validity of the proposed strategy presented in the previous section. The projections composed by the Shepp–Logan phantom image *e* in Figure 6a were reconstructed. The objective functions $D^{m}\left(e,z\left(n\right)\right)$ of *e* and z(n) from the WBIR and OSEM algorithms for n=0,1,2,…,60 are shown in Figure 11. Here, it can be seen that WBIR reduces the objective function much more than any OSEM does. The experimental result verified the strategy that the largest difference in the objective functions caused by a one-step iteration results in a more effective scheme from the viewpoint of total optimization. The time courses of SNR and SSIM plotted in Figure 12 show the same property.

**Figure 13:**
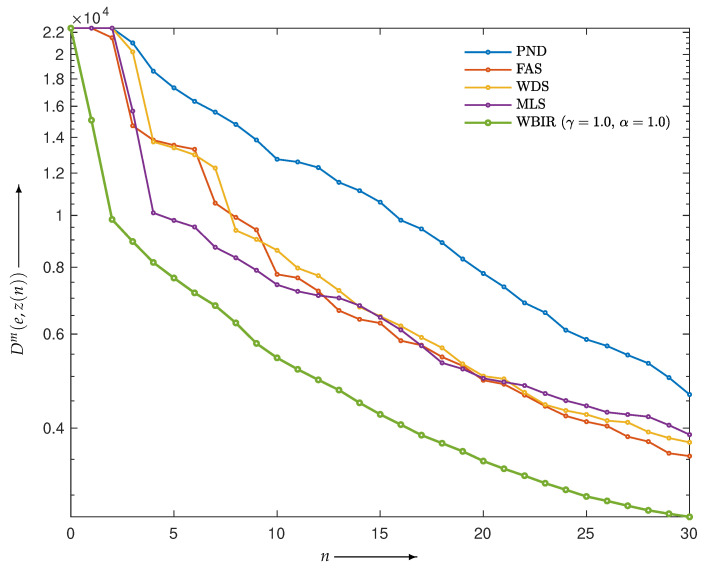
Objective functions $D^{m}\left(e,z\left(n\right)\right)$ for WBIR and OSEM algorithms by PND, FAS, WDS, and MLS at each iteration *n* = 0, 1, 2, …, 60 in experiment using chessboard phantom with noise-free projections.

**Figure 14:**
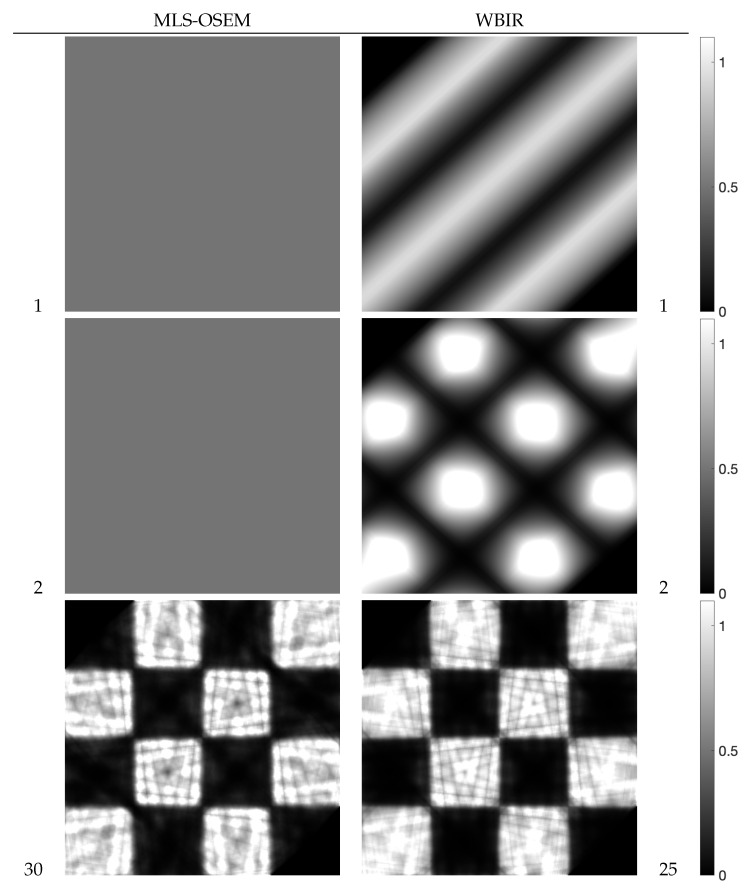
Reconstructed images at iterations denoted by the number beside each image for MLS-OSEM and WBIR in experiment using chessboard phantom. Thirty iterations by OSEM have almost the same computation time as 25 by WBIR.

**Figure 15:**
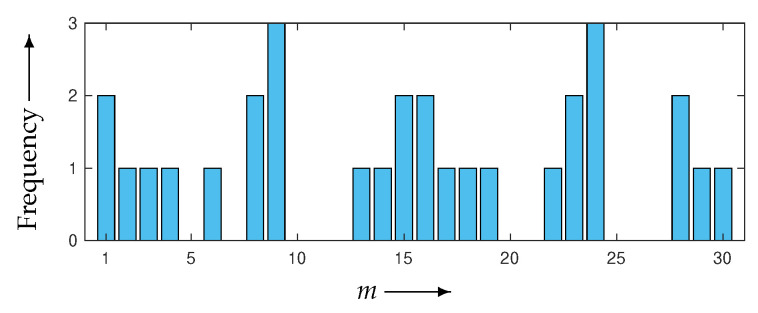
Frequency bar chart of subset indices *m* = 1, 2, …, 30 for WBIR after 30 iterations in experiment using chessboard phantom.

Next, we examined the effectiveness of the proposed WBIR algorithm in which the shape information in a phantom image is incorporated into the subset selection scheme. For this purpose, the phantom of a chessboard pattern image in Figure 6b was used. Because projections of $0^{\circ }$ and $90^{\circ }$ views for the chessboard are flat and identical to the corresponding forward projections of the constant initial states, as Guan and Gordon [19] indicated, an OS algorithm using MLS that starts ordering from the same two views reconstructs nothing. The resulting time courses of the objective functions are shown in Figure 13. We can see that WBIR has a rather better performance in, especially, the first and second iterations relative to the OSEM algorithms with PND, FAS, WDS, and MLS. Although this phantom shape and initial angle amount to a worst case for OS schemes, as shown in Table 2, WBIR dynamically selects the initial $132^{\circ }$ and $48^{\circ }$ views, which are approximations of $135^{\circ }$ and $45^{\circ }$, respectively, and are better choices with which to start the reconstruction for this shape of phantom. The effectiveness of the dynamic selection using the weeding function is visually confirmed by the reconstructed images shown in Figure 14. By comparing the images obtained from OSEM and WBIR, the iterative algorithm that reduces the objective function at every step as much as possible improves the quality of images not only in the initial steps but also in the last iterations more than uniform use of subsets as in Figure 15. It is interesting that some subsets are unnecessary for a high-quality reconstruction.

**Figure 16:**
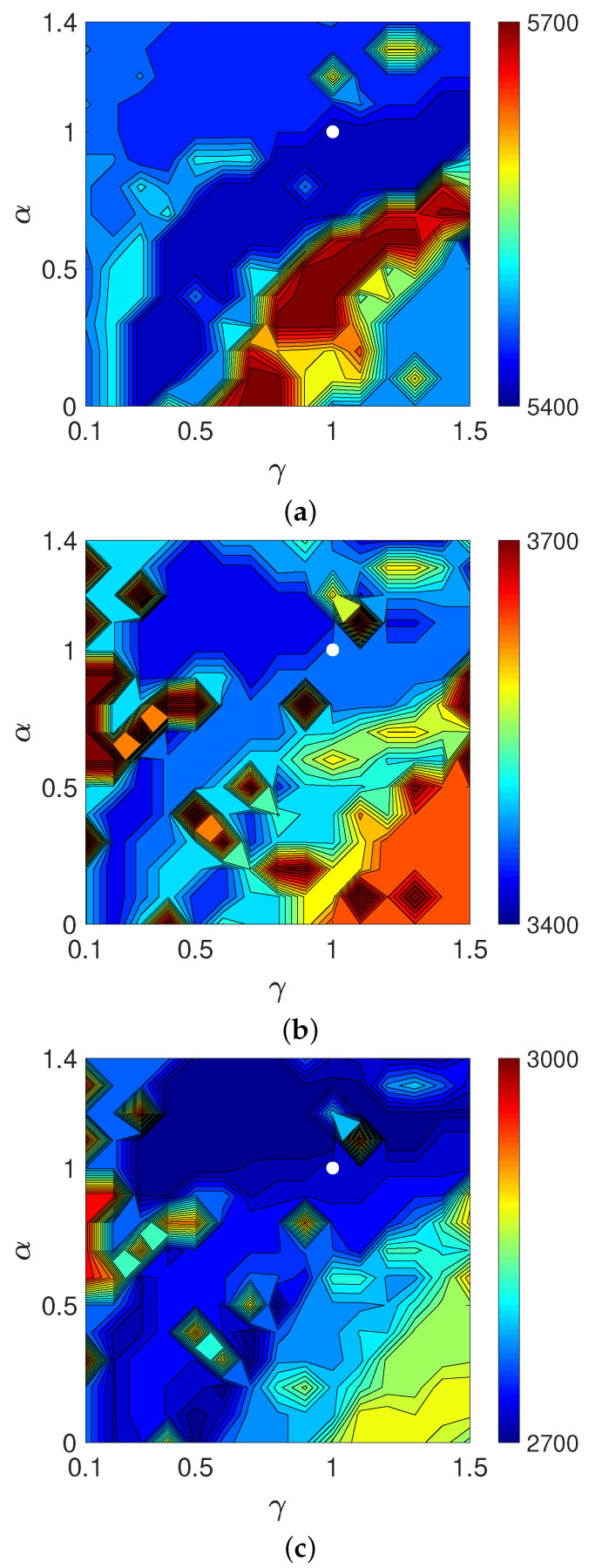
Contour plots of objective functions $\log_{10}\left(D^{m}\left(e,z\left(N\right)\right)\right)$ with *N* equal to (**a**) 10, (**b**) 20, and (**c**) 30 in experiment using noise-free projection. The white dot indicates the position of (γ,α)=(1,1).

Finally, we examined the validity of the weeding function in Equation (18) by replacing the estimating function ${\mathrm{EP}}_{1,1}$ defined as usual in Equation (22) with ${\mathrm{EP}}_{\gamma ,\alpha }$ and varied the parameters γ and α. Figure 16 shows contour plots of the objective functions on a logarithmic scale, $\log_{10}\left(D^{m}\left(e,z\left(N\right)\right)\right)$ for N=10, 20, and 30, in the parameter plane (γ,α) for MLEM-based WBIR with noise-free projections. The parameters γ and α were sampled from 0.1 to 1.5 and 0 to 1.4 with a sampling interval of 0.1, respectively. Though the parameter regions in which the values of the objective function are lowest vary depending on the iteration number, the parameter (γ,α)=(1,1) is a good common setting.

**Figure 17:**
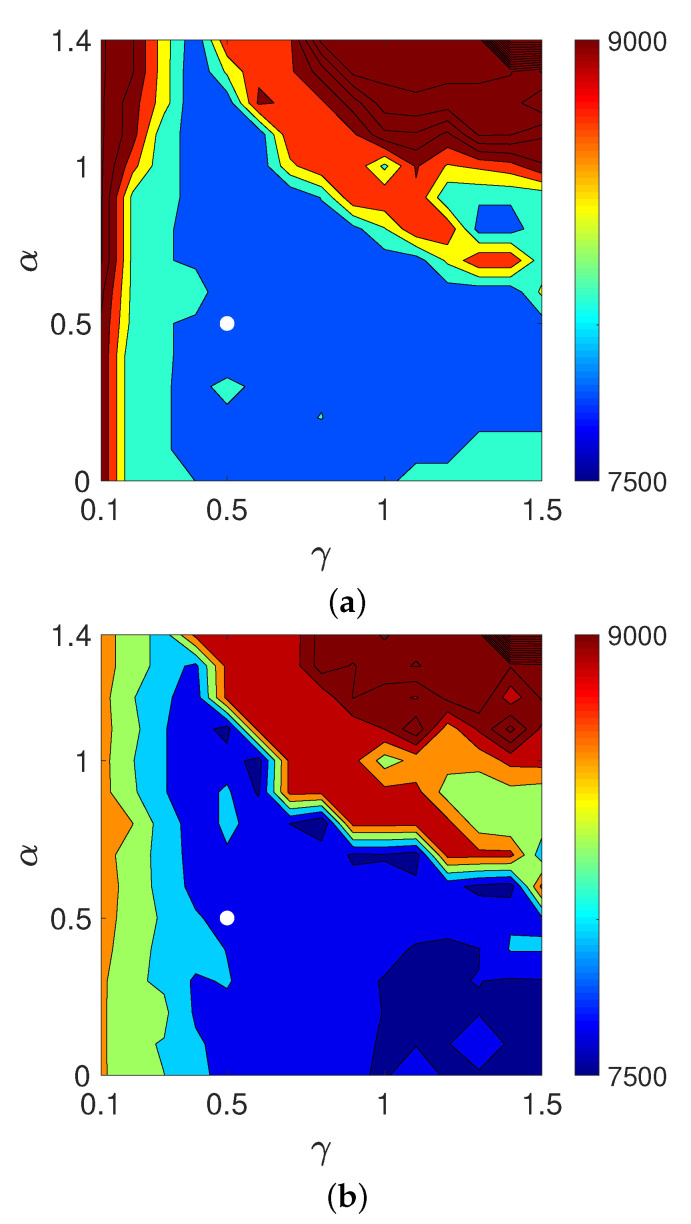

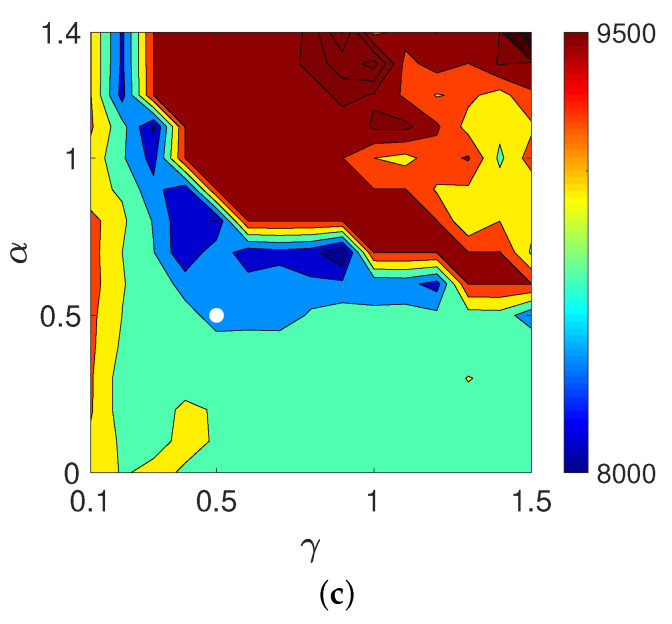
Contour plots of objective functions $\log_{10}\left(D^{m}\left(e,z\left(N\right)\right)\right)$ with *N* equal to (**a**) 10, (**b**) 20, and (**c**) 30 in experiment with noisy projection. The white dot indicates the position of (γ,α)=(0.5,0.5).

**Figure 18:**
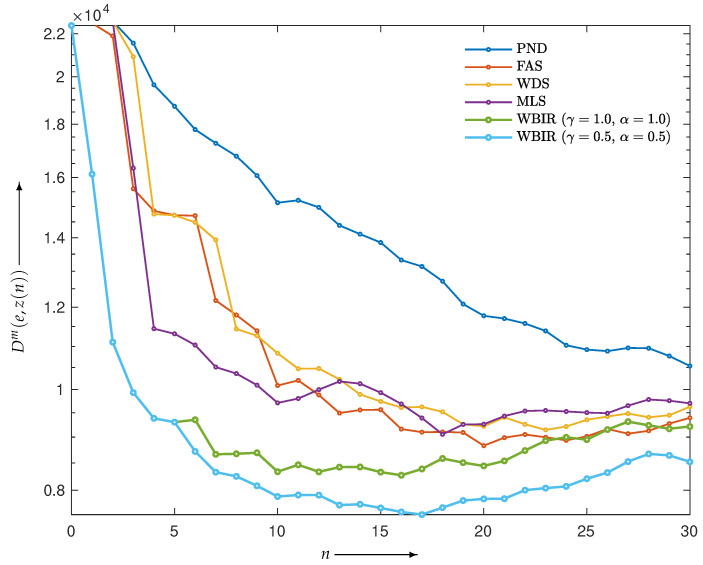
Objective functions $D^{m}\left(e,z\left(n\right)\right)$ for WBIR and OSEM algorithms by PND, FAS, WDS, and MLS at each iteration *n* = 0, 1, 2, …, 60 in experiment using chessboard phantom with noisy projections.

However, because the estimating function ${\mathrm{EP}}_{1,1}$ or equivalently the KL-divergence is not robust against outliers, it should be modified to deal with cases where the projection data are noisy. To search for an effective estimating function in ${\mathrm{EP}}_{\gamma ,\alpha }$, which is a family of extended power-divergence measures, we performed an experiment using projections in Equation (1) with δ as white Gaussian noise such that the signal-to-noise ratio was 20 dB. We made contour plots of the objective function in the (γ,α) plane. As shown in Figure 17, the pairs roughly around (0.5,0.5) give mostly lower values of the objective function for any iteration number. Using the divergence measure ${\mathrm{EP}}_{0.5,0.5}$ as an estimating function makes the reconstruction more robust to noise. Indeed, the resulting time courses of the objective functions plotted in Figure 18 indicate that the WBIR algorithm using the weeding function with ${\mathrm{EP}}_{0.5,0.5}$ outperforms the one with ${\mathrm{EP}}_{1,1}$.

## 5. Conclusions

Our block-iterative reconstruction, named WBIR, selects subsets of projections dynamically for solving the inverse problem of tomographic image reconstruction from sparse projection views on the basis of an estimating function constructed by using an extended power-divergence measure for decreasing the objective function as much as possible. We gave a unified proposition of an inequality related to the difference between the objective functions caused by a one-step iteration as a theoretical basis of the proposed optimization strategy. Theoretical analyses and numerical experiments showed that nonuniform and sparse use of projection views leads to a high-quality image reconstruction and that an ordered subset is not the most effective for block-iterative reconstruction. The two-parameter class of extended power-divergence measures is the key to estimating an effective decrease in the objective function and plays a significant role in constructing a robust algorithm against noise. An effective method of choosing parameters should be investigated as a way of improving the proposed algorithm.

The WBIR algorithm can be used for solving linear tomographic inverse problems in various areas besides biomedical and industrial imaging and is effective when the number of available measured projections is limited. However, its performance depends on, in terms of the meaning of decreasing the objective function and computing the estimating function, the sparseness relative to the dimension of the system variables. This is another issue to be explored in future studies.

## Figures and Tables

**Table 1:** Subset indices corresponding to the ten largest elements of ${\left\{\mathrm{LHS}(m)\right\}}_{m=1}^{M}$ (upper row) and ${\left\{\mathrm{RHS}(m)\right\}}_{m=1}^{M}$ (lower row) obtained by SART, MLEM, and MART-based BI reconstruction algorithms.

Method	Subset Indices									
BI-SART	17	15	30	2	18	14	29	3	16	19
	17	15	30	2	18	14	29	3	19	13
BI-MLEM	17	15	2	30	16	18	14	1	29	3
	17	15	2	16	30	18	14	1	29	3
BI-MART	17	15	2	30	18	14	16	29	3	1
	17	15	2	16	30	18	14	1	29	3

**Table 2:** Subset indices (upper row) and angles (lower row) of ten initial views for reconstructing chessboard phantom using MLS-OSEM and WBIR.

Method	Subset Indices and Angles									
MLS-OSEM	1	16	9	24	5	20	12	27	3	18
	0${}^{\circ }$	90${}^{\circ }$	48${}^{\circ }$	138${}^{\circ }$	24${}^{\circ }$	114${}^{\circ }$	66${}^{\circ }$	156${}^{\circ }$	12${}^{\circ }$	102${}^{\circ }$
WBIR	23	9	19	2	13	30	16	24	8	1
	132${}^{\circ }$	48${}^{\circ }$	108${}^{\circ }$	6${}^{\circ }$	72${}^{\circ }$	174${}^{\circ }$	90${}^{\circ }$	138${}^{\circ }$	42${}^{\circ }$	0${}^{\circ }$

## Data Availability

All data used to support the findings of this study are included within the article.
